# Supplementary material for: Unique Features of the m6A Methylome and Its Response to Salt Stress in the Roots of Sugar Beet (Beta vulgaris)
Source: Int J Mol Sci. 2023 Jul 19;24(14):11659. doi: 10.3390/ijms241411659 (PMC10380635; doi:10.3390/ijms241411659)
Supplement: Supplementary file 1 [file ijms-24-11659-s001.zip › Figures S1 and S2.pdf]

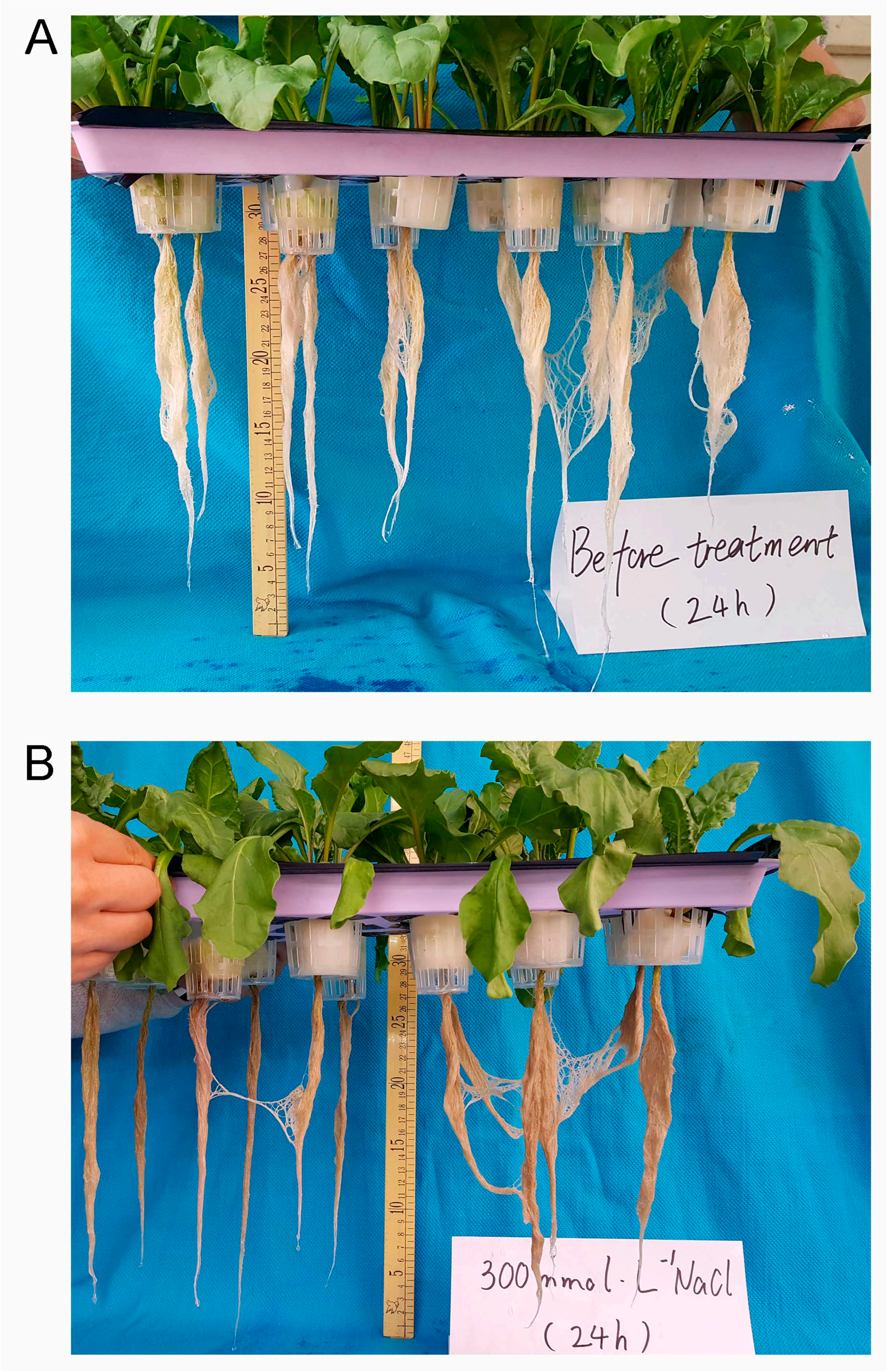

Figure S1: Changes in seedling roots before (A) and after (B) salt treatment.

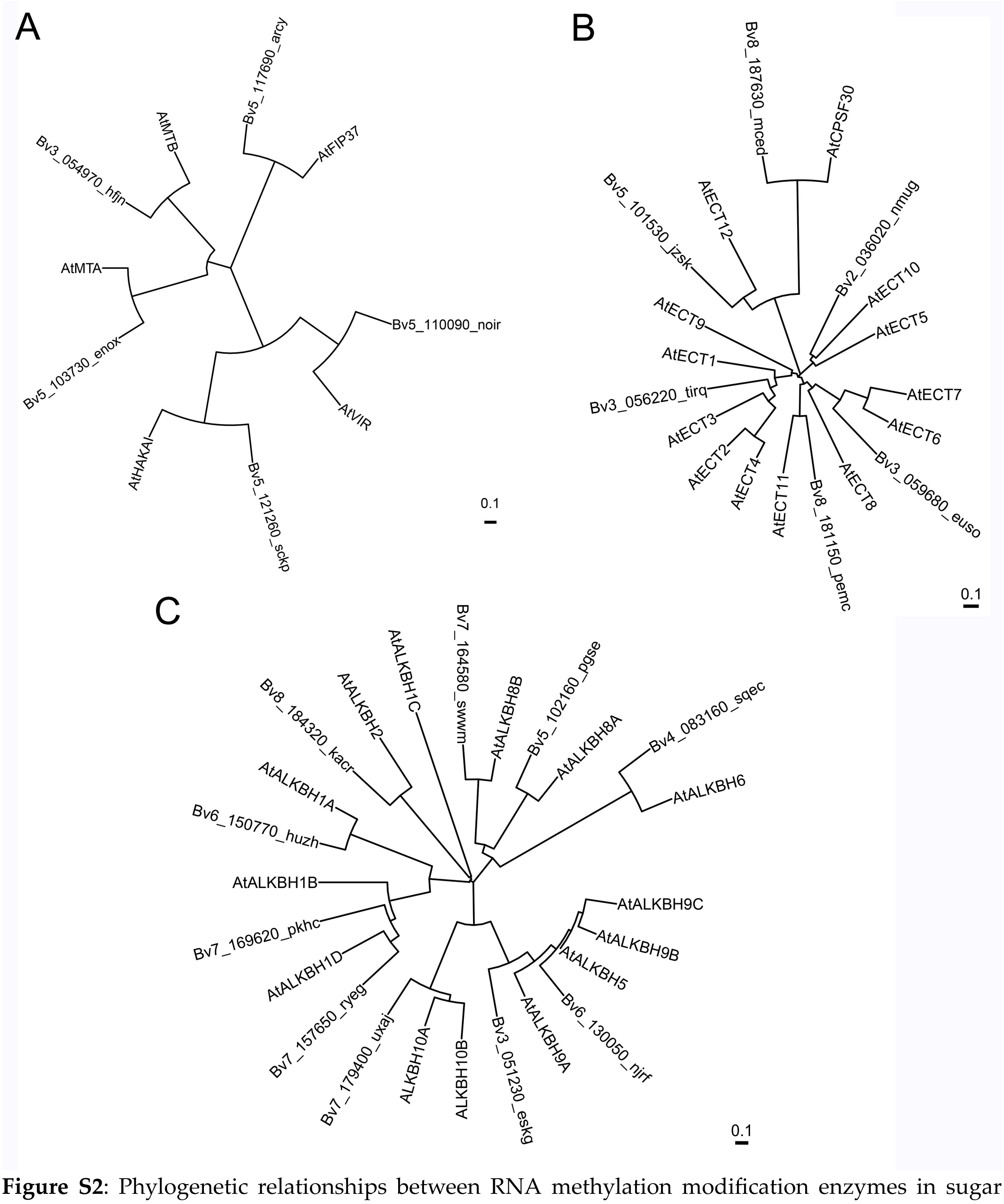

Figure S2: Phylogenetic relationships between RNA methylation modification enzymes in sugar beets and Arabidopsis thaliana. (A) transmethylase. (B) reading protein. (C) demethylase.
